# Supplementary material for: From sequence to enzyme mechanism using multi-label machine learning
Source: BMC Bioinformatics. 2014 May 19;15:150. doi: 10.1186/1471-2105-15-150 (PMC4229970; doi:10.1186/1471-2105-15-150)
Supplement: Additional file 2 — Java code of ml2db. Additional file ml2db_code.tar.gz contains the Java source code to run the multi-label machine learning experiments and save the results to database. The code’s Javadoc is included. [file 1471-2105-15-150-S2.zip › additional file 2/ml2db/ml2db/doc/main/ml2db/mulan/learn/crosseval/class-use/CrossExperimenter.html]

Uses of Class main.ml2db.mulan.learn.crosseval.CrossExperimenter


JavaScript is disabled on your browser.


- Overview
- Package
- Class
- Use
- Tree
- Deprecated
- Index
- Help

- Prev
- Next

- Frames
- No Frames

- All Classes

## Uses of Class main.ml2db.mulan.learn.crosseval.CrossExperimenter

- Packages that use CrossExperimenter

  | Package | Description |
  | --- | --- |
  |  |  |
  | --- | --- |
  | main.ml2db.al.learning |  |
  | main.ml2db.mulan.learn.crosseval |  |
  | main.ml2db.mulan.learn.evaluation |  |
  | main.ml2db.mulan.learn.traintest |  |
  | main.ml2db.mulan.predict |  |
  | test.ml2db.mulan.learn |  |
- - ### Uses of CrossExperimenter in main.ml2db.al.learning

    Subclasses of CrossExperimenter in main.ml2db.al.learning

    | Modifier and Type | Class and Description |
    | --- | --- |
    |  |  |
    | --- | --- |
    | `class` | `AlExperimenter` Class |
  - ### Uses of CrossExperimenter in main.ml2db.mulan.learn.crosseval

    Methods in main.ml2db.mulan.learn.crosseval that return CrossExperimenter

    | Modifier and Type | Method and Description |
    | --- | --- |
    |  |  |
    | --- | --- |
    | `CrossExperimenter` | CrossInputManager.`getExperiment()` |
    | `CrossExperimenter` | CrossOutputManager.`getExperimenter()` |

    Constructors in main.ml2db.mulan.learn.crosseval with parameters of type CrossExperimenter

    Constructor and Description

    `CrossEvaluator(CrossExperimenter experimenter,
    boolean strict)`

    `CrossInputManager(IdMultiLabelInstances trainSet,
    CrossExperimenter experiment)`

    Gets the instances as object.

    `CrossInputManager(int trainArffRecordId,
    DbManagers dbMan,
    CrossExperimenter crossExperimenter)`

    Gets the instances from database arff record.

    `CrossOutputManager(uk.ac.standrews.utils.main.database.DbManager resultsDbManager,
    java.lang.String resultsFolder,
    uk.ac.standrews.utils.main.database.Table table,
    CrossExperimenter experiment)`
  - ### Uses of CrossExperimenter in main.ml2db.mulan.learn.evaluation

    Methods in main.ml2db.mulan.learn.evaluation that return CrossExperimenter

    | Modifier and Type | Method and Description |
    | --- | --- |
    |  |  |
    | --- | --- |
    | `CrossExperimenter` | EvaluatorNoAUC.`getExperimenter()` |

    Constructors in main.ml2db.mulan.learn.evaluation with parameters of type CrossExperimenter

    Constructor and Description

    `EvaluatorNoAUC(CrossExperimenter experimenter,
    boolean strict)`
  - ### Uses of CrossExperimenter in main.ml2db.mulan.learn.traintest

    Subclasses of CrossExperimenter in main.ml2db.mulan.learn.traintest

    | Modifier and Type | Class and Description |
    | --- | --- |
    |  |  |
    | --- | --- |
    | `class` | `TrainTestExperimenter` Using Mulan (multi-label machine learning), this class initialises and executes Train-Test evaluation (two arff records are needed, for the training and testing files) (calling `TrainTestEvaluator`). |
    | `class` | `TrainTestExperimenterSerialized` Executes a train/test evaluation using a previously trained model saved on file. |
  - ### Uses of CrossExperimenter in main.ml2db.mulan.predict

    Constructors in main.ml2db.mulan.predict with parameters of type CrossExperimenter

    Constructor and Description

    `PredictionsSaver(CrossExperimenter crossExper,
    mulan.data.MultiLabelInstances train,
    mulan.data.MultiLabelInstances test,
    mulan.classifier.MultiLabelLearner trainedLearner,
    boolean isCrossEvaluation,
    boolean saveTrueNegatives,
    int fold)`

    Predictor hooked into a cross-evaluation experiment.
  - ### Uses of CrossExperimenter in test.ml2db.mulan.learn

    Methods in test.ml2db.mulan.learn that return CrossExperimenter

    | Modifier and Type | Method and Description |
    | --- | --- |
    |  |  |
    | --- | --- |
    | `static CrossExperimenter` | ExperimenterTest.`getMulanExperimenter(int arffId)` |
    | `static CrossExperimenter` | ExperimenterTest.`getMulanExperimenter(int arffId, java.lang.String resultsDbProps, java.lang.String arffRecordDb)` |
    | `static CrossExperimenter` | ExperimenterTest.`getMulanExperimenter2()` |
    | `static CrossExperimenter` | ExperimenterTest.`getMulanExperimenterFilterTrainset()` |

- Overview
- Package
- Class
- Use
- Tree
- Deprecated
- Index
- Help

- Prev
- Next

- Frames
- No Frames

- All Classes
